# Preclinical evaluation of dual PI3K-mTOR inhibitors and histone deacetylase inhibitors in head and neck squamous cell carcinoma

**DOI:** 10.1038/bjc.2011.495

**Published:** 2011-11-24

**Authors:** R B Erlich, Z Kherrouche, D Rickwood, L Endo-Munoz, S Cameron, A Dahler, M Hazar-Rethinam, L M de Long, K Wooley, A Guminski, N A Saunders

**Affiliations:** 1 Department of Epithelial Pathobiology Group, Epithelial Cancer Programme, University of Queensland Diamantina Institute, Princess Alexandra Hospital, 4th Floor, Building 1, R wing, Ipswich Road, Woolloongabba, QLD 4102, Australia; 2 Oncology Department, Princess Alexandra Hospital, Brisbane, QLD, Australia; 3 School of Biomedical Sciences, University of Queensland, St Lucia, Queensland, Australia

**Keywords:** squamous cell carcinoma, phosphoinositol 3 kinase inhibitor, histone deacetylase inhibitor, combination therapy

## Abstract

**Background:**

We examine the potential value of a series of clinically relevant PI3K-mTOR inhibitors alone, or in combination with histone deacetylase inhibitors, in a model of head and neck squamous cell carcinoma (HNSCC).

**Methods:**

Head and neck squamous cell carcinoma cell lines, human keratinocyte and HNSCC xenograft models were treated with histone deacetylase inhibitors (HDACIs) and new generation PI3K and dual PI3K-mTOR inhibitors either alone or in combination. Cell and tumour tissue viability and proliferation were then determined *in vitro* and *in vivo*.

**Results:**

Phosphatidylinositol-3-phosphate kinase, AKT and dual PI3K-mTOR inhibitors caused marked *in vitro* enhancement of cytotoxicity induced by HDACIs in HNSCC cancer cells. This effect correlates with AKT inhibition and is attenuated by expression of constitutively active AKT. Histone deacetylase inhibitor and phosphatidylinositol-3-phosphate kinase inhibitors (PI3KIs) inhibited tumour growth in xenograft models of HNSCC. Importantly, we observed intratumoural HDAC inhibition and PI3K inhibition as assessed by histone H3 acetylation status and phospho-AKT staining, respectively. However, we saw no evidence of improved efficacy with an HDACI/PI3KI combination.

**Interpretation:**

That PI3K and dual PI3K-mTOR inhibitors possess antitumour effect against HNSCC *in vivo*.

Head and neck squamous cell carcinoma (HNSCC) is the sixth most common cancer in the developed world with an annual incidence of >500 000 cases worldwide, representing 3.2% of all newly diagnosed cancers in the United States alone (Jemal *et al*, 2004; Mao *et al*, 2004). Although important advances in the surgical and radiological treatment of HNSCC have occurred in the last decades, these tumours are still associated with severe disease- and treatment-related morbidity and have a 5-year survival rate of ∼50% (Haddad and Shin, 2008). These figures indicate the need for new therapeutic approaches. In this regard, two new classes of anticancer agent, namely, histone deacetylase inhibitors (HDACIs) and phosphatidylinositol-3-phosphate kinase inhibitors (PI3KIs), may have potential as therapies for HNSCC.

Head and neck squamous cell carcinoma, like all cancers, are associated with multiple genetic defects, which have been linked to dysregulation of basic biological processes (Forastiere *et al*, 2001; Serewko *et al*, 2002; Wong *et al*, 2005; Haddad and Shin, 2008; Endo-Munoz *et al*, 2009). In particular, dysregulation of signal transduction is a common feature of these tumours. For example, aberrant signalling in HNSCCs involving the MAPK and PI3K-AKT pathways is well described (Amornphimoltham *et al*, 2004, 2005; Van Baal *et al*, 2006; Bussink *et al*, 2008). AKT activation frequently occurs in HNSCC because of PIK3CA mutations and AKT2 amplification (Pedrero *et al*, 2005). AKT activation is an early event in HNSCC progression and represents an independent prognostic marker of poor clinical outcome in tongue and oropharyngeal HNSCC (Massarelli *et al*, 2005; Yu *et al*, 2007a, 2007b). These data highlight the potential significance of targeting the PI3K/AKT signalling pathways in HNSCC (Van Baal *et al*, 2006; Bussink *et al*, 2008; Haddad and Shin, 2008).

Histone deacetylase inhibitors have shown promise as anticancer agents and are synergistic or additive with other antineoplastic treatments including radiation, chemotherapy, differentiation agents, epigenetic therapy and new targeted agents (Dowdy *et al*, 2006; Shen *et al*, 2007; Erlich *et al*, 2008). Of particular note, it was previously shown that HDACIs might modulate the PI3K and MAPK pathways (Rahmani *et al*, 2003a, 2005; Yu *et al*, 2005; Gao *et al*, 2006). Earlier studies from our laboratory and other groups indicated that a variety of HDACIs exhibit anticancer properties against squamous cell carcinomas (SCC) *in vitro* suggesting they may have use in a clinical setting (Saunders *et al*, 1999a, 1999b; Brinkmann *et al*, 2001; Gillenwater *et al*, 2007; Erlich *et al*, 2008). However, recent patient trials have shown that, as monotherapies, HDACIs had limited clinical potential for the treatment of HNSCC (Blumenschein *et al*, 2008; Erlich *et al*, 2008). Although it is possible to improve the therapeutic effects of HDACIs by combining them with other anticancer agents such as chemotherapy, irradiation, proteasome inhibitors, death receptor agonists and kinase inhibitors (reviewed in Frew *et al*, 2009) the potential of new therapeutic regimens for HNSCC based on the combination of HDACIs and targeted agents remains poorly investigated. In the present study we examine whether selective targeting of the PI3K-AKT and MAPK signalling pathways can improve the therapeutic potential of HDACIs in HNSCC cell lines and xenograft HNSCC models.

## Materials and methods

### Chemicals

SAHA (Vorinostat) was provided by Merck (Whitehouse Station, NJ, USA). LBH589 (Panobinostat), BEZ235, BKM120, BGT226 were all provided by Novartis (Basel, Switzerland). Valproic acid and *α*-tocopherol were purchased from Sigma (Sydney, NSW, Australia). Depsipeptide was obtained from Gloucester Pharmaceuticals (Cambridge, MA, USA). U0126, LY294002, wortmannin and AKT VIII were purchased from Cell Signalling (Danvers, MA, USA). ZVAD-fmk was purchased from Alexis Biochemicals (Exeter, UK). Cisplatin was purchased from DBL (Rowville, VIC, Australia). Some of the drug preparations were made in cell culture-grade dimethylsulphoxide (DMSO). The final concentrations of DMSO in the culture medium in all experiments were a maximum of 0.2% (v/v). Polyclonal antibodies recognising total AKT, phosphorylated AKT (Ser 473), phosphorylated p44/p42 MAPK, phosphorylated GSK3*β* and *α*/*β* tubulin were obtained from Cell Signalling. Polyclonal antibody recognising Erk2 was purchased from Santa Cruz (Santa Cruz, CA, USA). Polyclonal antibody recognising Myc was purchased from Upstate (Waltham, MA, USA). Peroxidase-conjugated anti-rabbit IgG secondary antibody was purchased from GE Healthcare (Chalfont, Bucks, UK). Chemicals and reagents were analytical grade or better.

### Treatments

In co-treatment assays, kinase inhibitors were added 10 min before the histone deacetylase inhibitors. Vitamin E and ZVAD-fmk were added 30 min before other treatments.

### Western blotting

Protein extractions and western blot assays were performed as previously described (Erlich *et al*, 2008). Membranes were incubated with the following primary antibodies: phospho-Erk 1 : 1000, phospho-AKT (S473) 1 : 1000, phospho-GSK3*β* 1 : 1000, Erk2 1 : 8000, AKT 1 : 5000, myc 1 : 2000, *α*/*β* tubulin 1 : 1000 and actin 1 : 8000.

### Maintenance of cells

Normal human keratinocytes (HKs) were isolated and cultured from neonatal foreskins following circumcision as previously described (Jones *et al*, 1997). SCC9, SCC25 and Cal27 tumour cell lines were grown and maintained as previously described (Erlich *et al*, 2008). Cell line validation has been previously reported (Poth *et al*, 2010).

### Single clone isolation

Single cell clones were prepared as previously described (Poth *et al*, 2010).

### Proliferation assays

BrDU incorporation measurements were performed with the Cell Proliferation assay kit (Roche, Nutley, NJ, USA, no. 11647229001) according to the manufacturer's protocol.

### Reactive oxygen species measurement

Cells were plated in six-well plates at 2.5 × 10^5^ cells per well. After distinct treatments, cells were harvested, washed twice with PBS, suspended in PBS with CM-H2DCFDA to a final concentration of 10 *μ*M, and incubated at 37°C for 20 min. ROS accumulation was measured by fluorescence intensity (FL-1, 530 nm) of 10 000 cells using a FACS Calibur flow cytometer (Becton Dickinson, North Ryde, NSW, Australia). Mean fluorescence intensity was obtained by histogram statistics using the CellQuest software (BD Biosciences, San Jose, CA, USA).

### Cytotoxicity and viability assays

Measurement of lactate dehydrogenase (LDH) release was performed with the CytoTox 96 Non-Radioactive Cytotoxicity Assay kit (Promega, Madison, WI, USA, no. G1780) according to manufacturer's protocol. Viability was assessed with the CellTiter 96 Aqueous Solution assay kit (Promega, no. G3580) according to manufacturer's protocol.

### Transfections

SCC25 cells were transfected using FuGENE 6 (Roche) according to manufacturer's protocol.

### Tumour immunohistochemistry

Avidin-biotin peroxidise procedure was used for immunostaining as previously described (Cameron *et al*, 2010). Primary antibodies used were as follows: phospho-AKT (1 : 100; Abcam, Cambridge, MA, USA), acetyl-histone H3 (1 : 2000), cleaved caspase 3 (1 : 250) or BrdU (1 : 100).

### BrdU and caspase 3 labelling quantification

For each experimental group, the percentage of BrdU-stained and active caspase 3-stained cells were assessed in several random areas using the NIS-Elements Br 3.1 software (Nikon, Melville, NY, USA). A minimum of 22 fields (area 142049.28 *μ*m^2^) were counted for each group.

### *In vivo* tumour studies

All animal experiments were approved by the Institutional Animal Ethics Committee. Six-week old female NOD-SCID mice were injected s.c. in the neck scruff with 2.5 × 10^5^ Cal27 or SCC25 cells. Groups of four mice received the following treatments when tumours were of approximately 0.4 cm^3^ volume: (i) vehicle only, (ii) LBH589 (30 mg kg^−1^ day^−1^ i.p.), (iii) BEZ235 (30 mg kg^−1^ day^−1^ p.o.), (iv) BGT226 (10 mg kg^−1^ day^−1^ p.o.), (v) BKM120 (7.5 mg kg^−1^ day^−1^ p.o), (vi) LBH589 (30 mg kg^−1^ day^−1^ i.p.)+BEZ235 (30 mg kg^−1^ day^−1^ p.o.), (vii) LBH589 (30 mg kg^−1^ day^−1^ i.p.)+BGT226 (10 mg kg^−1^ day^−1^ p.o.), (viii) LBH589 (30 mg kg^−1^ day^−1^ i.p.)+BKM120 (7.5 mg kg^−1^ day^−1^ p.o.). Stocks of LBH589 were prepared in DMSO (180 mM) and injectable solutions were prepared from this stock before injection. Stocks (stable for 1 week at 4°C) of BEZ235, BGT226 and BKM120 were prepared in 1-methyl-2-pyrrolidone (NMP, Fluka no. 69118, Castle Hill, NSW, Australia). Immediately before use, the stocks were diluted in PEG300 (Fluka no. 81160) (9 : 1 PEG:NMP) and administered by feeding tube (Becton Dickinson). Mice received daily treatments for 5 days per week over a 3-week treatment period. Tumour growth and animal weights were monitored for a period of up to 12 weeks. Animals were killed if tumour volumes exceeded 1 cm^3^. Three hours before killing the mice, they were administered the final dose and were injected (i.p.) with 20 *μ*l g^−1^ of a 10 mM stock BrdU.

### Statistical analysis

Data were analysed by Student's *t*-test when two groups are compared or ANOVA followed by *post-hoc* comparisons (Tukey's test) when multiple groups are compared.

## Results

### Vorinostat induces squamous cell carcinoma selective cytotoxicity

**Figure 1:**
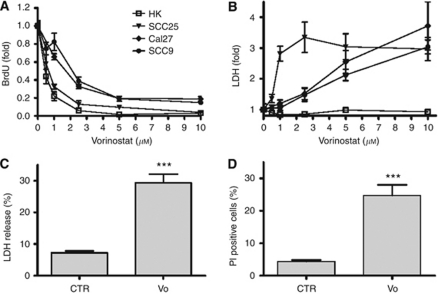
Vorinostat (vo) induces SCC cancer selective cytotoxicity. SCC cell lines (SCC25, Cal27, SCC9) and HKs were treated with varying concentrations of vofor 24 h. (**A**) BrdU incorporation and (**B**) cytotoxicity for three different HNSCC cell lines and HKs were determined as described in the text. Values are means±s.e. of two independent experiments performed in triplicate. (**C**, **D**) SCC25 cells were treated with vo (5 *μ*M) for 24 h and the LDH release (**C**) or PI staining (**D**) calculated as a percent of total. ^***^Indicates *P*⩽0.001 *vs* control (CTR). Values are mean±s.e.m. of at least three experiments performed in triplicate.

Following a 24-h treatment period, increasing concentrations of vorinostat (1–10 *μ*M) were able to inhibit proliferation of HNSCC cell lines and HKs in a dose-dependent manner (Figure 1A). Similarly, lactate dehydrogenase release assays showed that vorinostat induced cell death in a dose- and cell type-dependent manner in all tested cancer cell lines (Figure 1B). Importantly, vorinostat did not induce cell death in HKs even at 10 *μ*M (Figure 1B).

In contrast to the strong cytostatic effect observed in all cancer cell lines and HKs, the proportion of cancer cells affected by the cytocidal effects of vorinostat was much smaller. LDH release and PI staining assays showed that at maximal cytocidal doses (5 *μ*M) vorinostat induced cell death in no more than 30% of the SCC25 cell line (Figure 1C and D).

### Enhancement of vorinostat-induced cytotoxicity by PI3K inhibitors is associated with a persistent AKT inhibition

**Figure 2:**
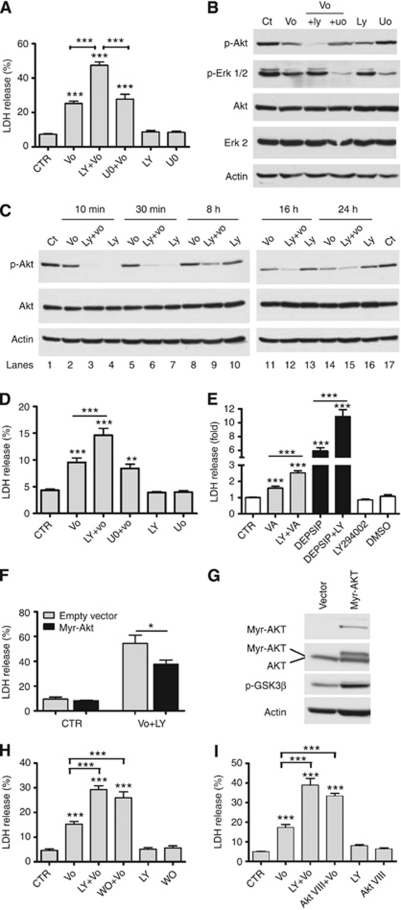
PI3K-AKT inhibitors enhancement of vorinostat (vo)-induced cytotoxicity is mediated by strong and durable AKT inhibition. (**A**) LDH viability assay for SCC25 cells treated with vehicle (CTR) or a maximal cytocidal dose of vo (5 *μ*M) alone or in combination with LY (10 *μ*M) or uo (10 *μ*M) for 24 h. (**B**, **C**) Western blots show lysates from SCC25 cells treated with vo (5 *μ*M) alone or in combination with LY (10 *μ*M) or uo (10 *μ*M) at distinct time points. (**D**) LDH viability assay for Cal27 cells treated with vehicle (CTR) or vo (5 *μ*M) alone or in combination with LY (10 *μ*M) or uo (10 *μ*M) for 24 h. (**E**) LDH viability assay for SCC25 cells treated with valproic acid (VA, 3 mM) or Depsipeptide (Depsip, 5 nM) alone or in combination with LY (10 *μ*M). (**F**) SCC25 cells were transfected with constitutively active AKT (myr-AKT, black boxes) or its corresponding empty vector (open boxes) and then left untreated (CTR) or were treated for 24 h with vo (5 *μ*M)+LY294002 (10 *μ*M, LY). Cell viability was then estimated by LDH release. (**G**) Western blot of lysates from SCC25 cells showing the expression of the myr-AKT and endogenous AKT in vector only and myr-AKT transfected cells. Phosphorylation of the AKT target GSK3*β* (p-GSK3*β*) are provided to confirm functional AKT activity. Western blot figures are representative of two independent experiments. (**H**) LDH viability assay for SCC25 cells treated with vo (5 *μ*M) alone or in combination with LY (10 *μ*M) or wortmannin (1 *μ*M). (**I**) Similar assay for cells treated with vo (5 *μ*M) alone or in combination with AKTVIII (10 *μ*M) for 24 h. LDH values presented as a percent of total LDH. Western blot figures are representative of at least three independent experiments. ^*^Indicates *P*⩽0.05. ^*^^*^Indicates *P*⩽0.01. ^***^Indicates *P*⩽0.001 *vs* CTR. Values presented as mean±s.e.m. of at least three independent experiments performed in triplicate.

MEK/ERK and PI3K/AKT pathways are deregulated in the majority of HNSCCs and HNSCC cell lines (Amornphimoltham *et al*, 2005; Massarelli *et al*, 2005; Pedrero *et al*, 2005; Van Baal *et al*, 2006; Yu *et al*, 2007a, 2007b; Bussink *et al*, 2008). Hence, we tested if inhibition of PI3K or MEK by LY294002 (LY) or U0126 (U0), respectively, would enhance the cytotoxicity of vorinostat. SCC25 cells were treated for 24 h with a maximal cytocidal dose of vorinostat (5 *μ*M) alone or in combination with LY (10 *μ*M) or U0 (10 *μ*M). Co-treatment with LY induced a marked increase in vorinostat-induced cytotoxicity (Figure 2A). In contrast, U0 co-treatment did not affect vorinostat-induced cytocidal effects. Treatment with LY or U0 alone did not induce cell death as compared with untreated cells (Figure 2A). Western blots of total lysates from SCC25 cells treated for 10 min with LY or UO alone or in combination with vorinostat and probed against phospho-AKT (S473) or phospho-p42/44 antibodies confirmed that LY and U0 selectively inhibited AKT and ERK activities, respectively (Supplementary Figure 1A).

We examined the activation status of AKT and ERK following exposure to vorinostat, LY, U0 or the combination following 24 h treatments (Figure 2B). Although prolonged treatment with vorinostat or the inhibitors alone caused a small inhibition of AKT and ERK activities, vorinostat treatment in combination with LY or U0 induced a pronounced inhibition of AKT and ERK, respectively. Therefore, we investigated the dynamics of AKT phosphorylation at distinct time points during the 24 h treatment (Figure 2C). In contrast to the effect of treatments with vorinostat or LY alone, AKT inhibition induced by the vorinostat/LY combination persisted throughout the 24 h of treatment. These data suggest that the enhancement of vorinostat cytotoxicity induced by co-treatment with LY correlates with a strong and persistent inhibition of AKT (S473) phosphorylation. An interesting observation was the ability of HDACI treatment to reduce AKT activity (Figure 2C). This has not been reported before but was consistent throughout our studies. The molecular basis for this observation is currently under investigation.

Similar to the SCC25 cells, we found that Cal 27 cells were sensitive to vorinostat-induced cell death and this effect was significantly increased by co-treatment with LY, but not U0 (Figure 2D). In addition, we treated SCC25 cells with cytocidal doses of the structurally dissimilar HDACIs, valproic acid or depsipeptide alone or in combination with LY. Similar to the effect observed with vorinostat co-treatments, cytotoxicity mediated by valproic acid and depsipeptide was markedly increased by LY co-treatment (Figure 2E). These data indicate that LY can enhance the cytotoxicity of a broad range of structurally unrelated HDACIs and these effects are conserved between different HNSCC cell lines.

To confirm that inhibition of AKT activity contributed to the enhanced cytocidal response induced by the HDACI/PI3K inhibitor combination, we generated stable transfectants of SCC25 cells with a constitutively active myc-tagged myristoylated AKT (myr-AKT), or with the corresponding ‘empty vector’. Expression of myr-AKT caused a significant attenuation of cytotoxicity induced by the combination treatment (Figure 2F). Western blot analysis of total lysates confirmed expression of myr-AKT and hyperactivation of the AKT pathway in transfected SCC25 cells as measured by GSK3*β* phosphorylation status, a well-established AKT target (Figure 2G). Next, we examined the effects of vorinostat in combination with Wortmannin, a PI3K inhibitor structurally unrelated to LY294002, or with an isoform-specific AKT 1/2 inhibitor (AKT VIII). Both combination treatments caused a significant increase in cell death and caused a persistent inhibition of S473 AKT phosphorylation (Supplementary Figure 1B) when compared with the effects of vorinostat alone. Treatment with Wortmannin (Figure 2H) or AKT VIII (Figure 2I) alone did not induce cell death in SCC25. These data indicate that inhibition of the AKT pathway alone may not be sufficient to invoke a complete cytotoxic response but may sensitise cells to a subsequent cytotoxic stimulus.

### Vorinostat/LY combination treatment does not induce cell death in normal human keratinocytes

Cytotoxicity assays showed that none of the combination treatments were able to induce substantial cell death in HKs (Supplementary Figure 2A). Moreover, in contrast to cancer cells, vorinostat/LY combination treatment for 24 h did not induce a persisting inhibition of AKT activity in HKs (Supplementary Figure 2B). The cyclin-dependent kinase inhibitor *CDKN1A* (encoding p21^WAF1/CIP1^) is upregulated by vorinostat and other HDACIs in several cancer cell lines, an effect that correlates with cell cycle arrest and has been suggested to induce protection from cell death mediated by HDACIs (Burgess *et al*, 2001, 2004). Vorinostat treatment was able to induce expression of p21^WAF1/CIP1^ in both SCC25 cells as well as HKs (Supplementary Figure 2C) indicating that vorinostat/LY treatment induces a cancer cell-selective inhibition of the AKT pathway.

### ROS generation correlates with enhanced caspase-dependent cytotoxicity induced by vorinostat/LY combination in SCC cells

Previous studies indicated that vorinostat and other HDACIs induce ROS accumulation in several cell types, and that this effect is relevant to vorinostat-induced cell death (Ruefli *et al*, 2001; Xu *et al*, 2006; Yu *et al*, 2007a, 2007b). In addition, PI3K inhibitors have been shown to potentiate peroxide accumulation induced by chemotherapy (Ramos *et al*, 2005). Therefore, we investigated the effect of LY treatment upon vorinostat-induced ROS accumulation. Vorinostat treatment induced accumulation of ROS in SCC cells and this effect was enhanced by co-treatment with LY (Supplementary Figure 3A). Treatment of SCC25 cells with LY alone resulted in little change in ROS levels. Pre-treatment with the anti-oxidant *α*-tocopherol (vitamin E) (1 mM) ablated the increase in ROS induced by vorinostat or vorinostat/LY treatments (Supplementary Figure 3B). In addition, *α*-tocopherol inhibited vorinostat or vorinostat/LY induced cytotoxicity in SCC25 cells (Supplementary Figure 3C).

As it was previously shown that HDACIs can induce caspase-dependent and -independent apoptosis as well as autophagy (Garcia-Morales *et al*, 2005), we tested the effect of the pan-caspase inhibitor ZVAD-FMK upon SCC25 cells treated with vorinostat or the vorinostat/LY combination. Regardless of the treatment, pan-caspase inhibition completely abrogated cell death (Supplementary Figure 3D).

### Clinically relevant PI3K-AKT-mTOR inhibitors enhance cancer cell specific cytotoxicity induced by LBH589

**Figure 3:**
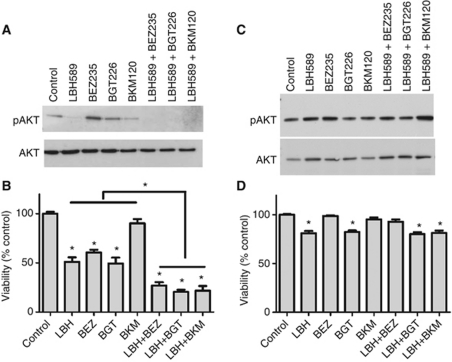
Clinically relevant PI3K-AKT-mTOR inhibitors enhance cancer cell specific cytotoxicity induced by LBH589. (**A**) Western blots show lysates from SCC25 cells treated for 48 h with LBH589 (300 nM), BEZ235 (300 nM), BKM120 (300 nM) and BGT226 (300 nM), alone in combinations. (**B**) Viability assay for SCC25 cells subjected to the same treatments as in (**A**). (**C**) Western blots show lysates from normal HKs subjected to the same treatments as in (**A**). (**D**) Viability assay for normal HKs cells subjected to the same treatments as in (**C**). Western blot figures are representative of three independent experiments. Values are means±s.e. of three independent experiments performed in triplicate. ^*^Indicates *P*<0.05.

We tested the effects of the HDACI, LBH589 (panobinostat) alone or in combination with the dual PI3K-mTOR inhibitors, BEZ235 and BGT226, or the PI3K inhibitor, BKM120. These new generation inhibitors have favourable pharmacokinetic profiles and are currently undergoing phase I/II clinical trials (Engelman, 2009; Tan *et al*, 2010). LBH589 causes a dose-dependent hyperacetylation of histone H3 in SCC25 cells and BEZ235, BGT226 or BKM120 caused a dose-dependent inhibition of AKT phosphorylation (S473) (Supplementary Figure 4A–D). Furthermore, LBH589, BEZ235, BGT226 or BKM120 caused a dose-dependent inhibition of proliferation and viability in SCC25 and Cal27 cells (Supplementary Figure 5). Similar to our earlier experiments, persistent inhibition of AKT phosphorylation was evident in SCC25 cells treated with the LBH589/PI3KI combinations (Figure 3A). Interestingly, LBH589 induced a persistent inhibition of phospho-AKT whereas none of the PI3Ks alone did (Figure 3A). Confirming our previous results, cytotoxicity induced by LBH589 was always enhanced by co-treatment with PI3K inhibitors in SCC25 cells (Figure 3B) and Cal27 cells (Supplementary Figure 6). Although absolute drug responses differed between SCC25 cells (Figure 3B) and Cal27 cells (Supplementary Figure 6) there was clear evidence that the HDACI/PI3KI combination was more effective than the drugs given individually. For example, the PI3K-specific inhibitor, BKM120, was not sufficient to cause cytotoxicity alone in SCC25 cells, whereas the dual PI3K-mTOR inhibitors, BGT226 and BEZ235, were able to induce cytotoxicity when given alone despite the lack of persistent phospho-AKT inhibition (Figure 3B). In contrast, the Cal27 cells responded equally well to all the PI3KIs (Supplementary Figure 6). In addition, treatment of HKs with LBH589 and BGT226 alone or LBH589 in combination with BGT226 or BKM120 had no effect on phospho-AKT levels (Figure 3C) and induced a small decrease in the viability of HKs (Figure 3D), HNSCC cells were much more sensitive to the cytocidal effects of these drugs alone or in combination (Figure 3D and Supplementary Figure 6).

### Antitumour properties of LBH589 and PI3K/AKT/mTOR inhibitors in a xenotransplant model of HNSCC

**Figure 4:**
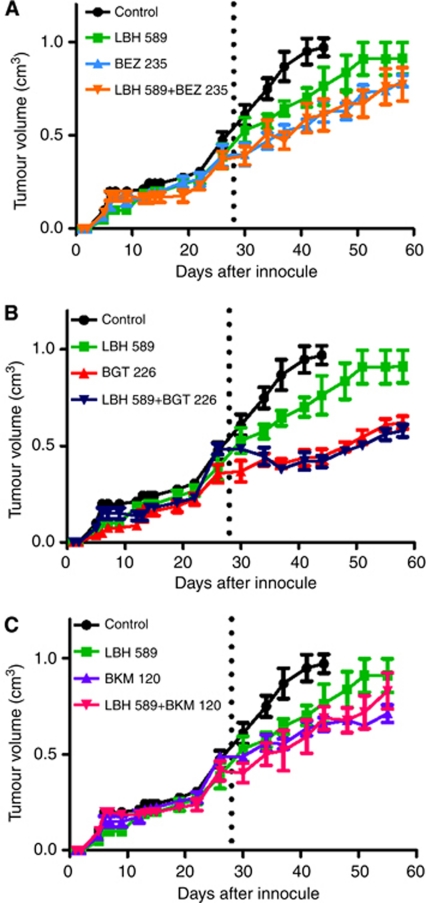
Antitumour properties of LBH589 and PI3K/mTOR/AKT inhibitors in a xenotransplant model of HNSCC. (**A**–**C**) Six week old NOD/SCID mice (groups of four) were injected with 2.5 × 10^5^ Cal27 cells on day 0. 28 days after injection of cells mice were treated with (i) vehicle only, (ii) LBH589 (30 mg kg ^−1^day^−1^ i.p.), (iii) BEZ235 (30 mg kg ^−1^day^−1^ p.o.), (iv) BGT226 (10 mg kg ^−1^day^−1^ p.o.), (v) BKM120 (7.5 mg kg ^−1^day^−1^ p.o), (vi) LBH589 (30 mg kg ^−1^day^−1^ i.p.)+BEZ235 (30 mg kg ^−1^day^−1^ p.o.), (vii) LBH589 (30 mg kg ^−1^day^−1^ i.p.)+BGT226 (10 mg kg ^−1^day^−1^ p.o.), (viii) LBH589 (30 mg kg ^−1^day^−1^ i.p.)+BKM120 (7.5 mg kg ^−1^day^−1^ p.o.). Tumour volume was measured over a period of 60 days post-tumour inoculation or until a volume of 1 cm^3^ was reached. Data presented as mean±s.e.m. of individual measurements from four mice per group. Dotted line indicates beginning of treatment for distinct groups.

**Figure 5:**
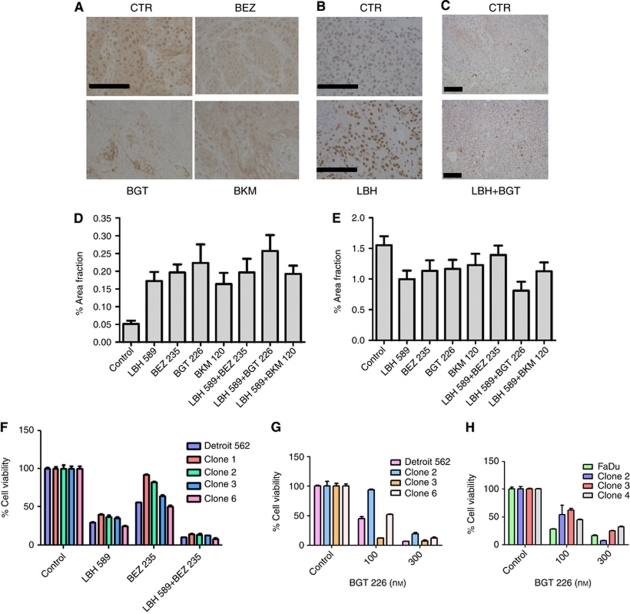
Assessment of the effects of PI3K inhibitors and HDACIs in tumour tissue. Representative figures shows immunostaining for (**A**) p-AKT, (**B**) acetyl-histone H3 and (**C**) active caspase 3 (bars=100 *μ*m). (**D**) Active caspase 3 labelling quantification and (**E**) BrdU labelling quantification in tumours derived from mice subjected to treatment with BEZ235, BGT226 and BKM120 alone or in combination with LBH589. (**F**) Viability assay following treatment of the parental SCC cell line Detroit and distinct clonal variants with LBH589 (300 nM), BEZ235 (300 nM) or both for 48 h. (**G**) Viability assay following treatment of the parental Detroit 562 SCC cell line and distinct clonal variants with BGT226 at 0, 100 or 300 nM for 48 h (**H**) Viability assay following treatment of the parental Fadu SCC cell line and distinct clonal variants with BGT226 at 0, 100 or 300 nM for 48 h. Values for (**D**) and (**E**) are means±s.e. for at least three independently treated mouse tumours. Values for (**F**) and (**G**) are means±s.e. from triplicate determinations.

The antitumour effect of the different LBH589/PI3K inhibitor combinations was analysed in xenograft models in NOD/SCID mice. Tumour growth was monitored until a mass of approximately 0.4 cm^3^ was palpable (28 and 48 days post tumour injection for Cal27 cells and SCC25 cells, respectively) at which time the animals were sham-treated or treated with LBH589, BEZ235, BGT226 and BKM120 alone or in combinations of LBH589 with each of the inhibitors (Figure 4A–C and Supplementary Figure 7). Tumour growth rate was reduced by treatment with the HDACI or PI3KIs alone. PI3K inhibitors given as mono-therapies were more effective than LBH589 alone in Cal27 tumours (Figure 4). Consistent with the *in vitro* data (Figure 3B and Supplementary Figure 6) the antitumour effect of the drugs and drug combinations was less substantial in the SCC 25 cells (Supplementary Figure 7) compared with Cal27 cells (Figure 4). The combination of LBH589 with any of the PI3K inhibitors did not invoke greater tumour growth control compared with the effects of BEZ235, BGT226 or BKM120 alone (Figure 4 and Supplementary Figure 7). We examined the pharmacodynamics of the HDACI and PI3KIs in Cal27 tumours at the end of the study. BEZ235, BGT226 and BKM120 reduced overall AKT phosphorylation levels (S473) and, in particular reduced nuclear phospho-AKT levels in Cal27 cells (Figure 5A). However, the PI3KIs did not cause complete inhibition of AKT activity (Figure 5A). LBH589 treatment induced an increase in histone H3 acetylation in the tumours (Figure 5B). These data indicated that the HDACIs and PI3K inhibitors were having the predicted pharmacological effect on the tumours. We observed a 3–5-fold increase in caspase 3 activation in tumour tissue following treatment with PI3K inhibitors alone or in combination with LBH589 (Figure 5C and D). BrdU incorporation assays indicated a modest decrease in tumour proliferation following the different treatments (Figure 5E). These data suggest the major pharmacological action of the PI3KIs is cytotoxic rather than cytostatic.

The *in vitro* and *in vivo* data suggest that total tumour ablation may be compromised by the existence of PI3KI- or HDACI-resistant subpopulations of cells in the various cell lines. This would result in the expansion of drug-resistant clonal variants during the 4-week drug treatment. Therefore, we examined the effect of LBH589 or BEZ235 treatment on cell viability of clonal variants of the Detroit 562 HNSCC cell line (Cameron *et al*, 2010; Poth *et al*, 2010) (Figure 5F). The results indicate that some clones are sensitive to BEZ235 whereas other clones are insensitive (Figure 5F). We went on to show that the clone-specific sensitivity also occurred with BGT226 in Detroit (Figure 5G) and Fadu (Figure 5H) cells but could be overcome by increasing the dose of PI3KI. In contrast, sensitivity to HDACI was similar between variants of the Detroit 562 cells (Figure 5F) indicating that the variation in PI3KI sensitivity between clonal variants of the different SCC cell lines was selective and did not reflect a general defect in cytotoxic response. These data indicate that clonal variants exist, *in vitro*, within the Fadu and Detroit 562 cell lines that differ in their sensitivity to PI3KIs.

## Discussion

In this manuscript we show that a series of PI3K inhibitors and an HDAC inhibitor are pharmacologically active and display cancer cell selective activity against xenotransplant models of HNSCC. The *in vitro* and *in vivo* antitumour activity of the HDACIs and the PI3KIs highlight several significant properties of these drugs. Firstly, all the drugs reduced tumour growth and increased tumour cell death when administered alone or in combination. Secondly, the drug combination appeared to work via AKT-dependent and AKT-independent pathways. Finally, we report that dose-dependent insensitivity to PI3KIs is present in some clonal variants of HNSCC *in vitro*.

The present study reports, for the first time, on the antitumour effects of PI3KIs delivered as monotherapies or in combination with HDACIs in models of HNSCC. HDACIs had modest antitumour effects *in vivo* whereas the PI3KIs, as a class, displayed encouraging antitumour activity. However, our *in vitro* and *in vivo* studies also indicated that subpopulations of cells exist within established HNSCC cell lines that differ in their sensitivity to HDACIs or PI3KIs and could limit the curative potential of PI3KIs or HDACIs as monotherapies. Significantly, our *in vitro* data suggest that the biological basis for insensitivity to HDACIs and PI3KIs differs. For example, analysis of clonal variants, *in vitro*, indicates that sensitivity to HDACIs is shared by all clonal variants within cell lines indicating that sensitivity is not dictated by heritable genetic/epigenetic differences between variants but can be attributed to physiological differences within the cell populations at any given time such as, cell cycle state or metabolic state. In contrast, varying sensitivity to PI3KIs is determined by heritable genetic/epigenetic differences between clonal variants within established cell lines. It is noteworthy that all HNSCC clones were sensitive to PI3KI at high doses. Had completely insensitive variants existed it would have manifest as an accelerated repopulation of the tumour following an initial treatment phase as has been observed by the emergence of resistance to agents such as the V600E-specific b-Raf inhibitors in melanoma (Villanueva *et al*, 2010) or the EGFRvIII inhibitors in glioma (Sampson *et al*, 2010). However, we observed a generalised, yet incomplete, antitumour response to PI3KI treatment, which would be consistent with a submaximal dose of PI3KI being achieved *in vivo*. With respect to the presence of clonal variants within tumours, we have recently shown that HNSCC cell lines contain clonal variants that differ in their transcriptomic signature, their tumourogenic potential and their sensitivity to cisplatin (Cameron *et al*, 2010; Poth *et al*, 2010). Significantly, we have also shown that these clonal variants exist in human SCCs *in situ* (Cameron *et al*, 2010). Thus, there is evidence that clonal variants with differing drug sensitivities may exist, which could impact on the effectiveness of molecular targeted therapies.

It could be argued that HDACI's are well tolerated *in vivo* and hence it may be possible that improved therapeutic effects could be seen with increased doses of HDACI. This would seem unlikely because our data clearly shows that the HDACI inhibits tumour HDAC activity (Figure 5). Moreover, data from clinical trials indicates that HDACIs have weak antitumour activity against solid tumours in patients (Blumenschein *et al*, 2008; Erlich *et al*, 2008). Thus, the *in vitro* and *in vivo* data would suggest that HDACIs have weak antitumour activity *in vivo* against HNSCC cells. The molecular basis for the resistance/sensitivity to HDACI remains unclear.

Although the PI3KIs showed marked antitumour activity *in vivo* there was still evidence of evasion of the cytotoxic effects by some tumour cells. Similar to HDACIs, the PI3KIs were being administered at doses sufficient to cause pharmacological inhibition of AKT (Figure 5). However, it is likely that the dose achieved was not sufficient to cause ablation of sensitive and insensitive clonal variants within the tumour. The PI3KIs were already being administered at the maximum tolerated dose (data not shown) and therefore it would not be possible to increase pharmacological activity of the PI3KIs in our xenotransplant model. Thus, it remains likely that the dose-limiting metabolic toxicity of the pan-PI3KIs prevented us from achieving maximal effective drug concentration in the xenotransplants. This situation is unlikely to occur in humans where the metabolic toxicities are likely to be clinically manageable allowing higher dosing schedules.

It is important to note that PI3KI treatment of HNSCC cell lines resulted in transient but complete inhibition of phospho-AKT indicating that all the cells are sensitive to the PI3K inhibitory activity. These data suggest that the differing degrees of sensitivity to PI3KIs may be because of defects residing downstream of AKT and/or because of parallel independent survival pathways that antagonise the cytocidal effects of the PI3KI. Indeed, recent studies have shown that cells with different mutations/amplifications in key steps of the PI3K-AKT pathway display differing sensitivity to BEZ235 (Brachmann *et al*, 2009). Thus, the overall sensitivity of solid tumours will be dictated by the relative proportions of variants in which sensitivity or insensitivity to the cytocidal/cytostatic, effects of PI3KI exist. The selective loss of signalling pathways within the SCC cell lines has been reported by us previously with respect to TGF*β*1-mediated signalling and growth inhibition (Dahler *et al*, 2001) and IFN*γ*-mediated growth inhibition and signalling (Saunders *et al*, 1999a, 1999b).

Our *in vitro* studies indicate that the mechanistic basis for HDACI and PI3KI action is likely to be tumour cell type-specific. For instance, different combination treatments involving HDACIs have been shown to modulate the PI3K-AKT and MAPK pathways (Rahmani *et al*, 2003a, 2005; Yu *et al*, 2005; Gao *et al*, 2006). Rahmani *et al* (2003b) demonstrated that inhibition of PI3K sensitised human leukaemia cells to various HDACIs. Interestingly, in this model HDACI treatment caused AKT activation and the cytotoxic sensitisation caused by HDACI/LY combination treatments were mediated through inactivation of MAPK, rather than AKT inhibition. In contrast, in our experimental model, inhibition of ERK activity following U0126 treatment in combination with vorinostat did not induce cell death. Also, in contrast to leukaemia cells, vorinostat did not cause activation of AKT in HNSCC cells *in vitro* or *in vivo*. Thus, the mechanism of action of drugs such as PI3KIs and HDACIs are likely to differ between tumour cell models.

In the present study, we show that the cytocidal effects of HDACIs and PI3KIs involve overlap between AKT-dependent and AKT-independent pathways. With regards to AKT-dependent cytotoxicity we showed that HDACIs induce cell death in HNSCC cell lines that was associated with a modest downregulation of AKT in SCC25 cells. Addition of a PI3KI to the HDACI enhanced cytotoxicity markedly and resulted in a profound, persistent and total ablation of AKT phosphorylation during the 24-h treatment period. In contrast, HKs failed to show persistent inhibition of AKT in response to PI3KI+HDACI and were insensitive to the cytocidal actions of the combination. Finally, enforced expression of constitutively active myr-AKT significantly attenuated cell death induced by HDACI/PI3KI treatment in SCC25 cells indicating that AKT inhibition contributes, at least in part, to the cytocidal action of this combination.

Although AKT inhibition clearly contributes to the HDACI and PI3KI-mediated cytotoxicity, it cannot explain all the cytocidal effects observed in this study. For instance, increased cytotoxicity was observed in response to HDACI or PI3KI treatment despite them being used individually at maximal doses *in vitro*. This indicates that the combination activates multiple independent cytotoxic pathways and that AKT inhibition may be required, but not sufficient, to invoke a complete cytotoxic response.
