# Supplementary material for: Preclinical evaluation of dual PI3K-mTOR inhibitors and histone deacetylase inhibitors in head and neck squamous cell carcinoma
Source: Br J Cancer. 2011 Nov 24;106(1):107–15. doi: 10.1038/bjc.2011.495 (PMC3251846; doi:10.1038/bjc.2011.495)
Supplement: Supplementary Figures [file bjc2011495x1.doc]

***Supplementary figure 1.*** Western blots show lysates from SCC25 subjected to the following treatments: (A) 10 min with vorinostat (Vo, 5 µM) alone or in combination with LY 294002(LY, 10 µM) or U0126 (U0, 10 µM); (B) 24 h with vorinostat (Vo, 5 µM) alone or in combination with Wortmmanin (wo; 1 µM) or AKTVIII (AKTI; 10 µM). Membranes were probed against phospho-AKT (S473), AKT, phospho-ERK1/2, ERK2 and actin antibodies. Figures are representative of three independent experiments with similar results.

***Supplementary figure 2. Vorinostat/LY combination treatment does not induce cell death in normal human keratinocytes****.* **(A)** LDH viability assay for HKs treated with vorinostat (5 µM) alone or in combination with LY294002 (10 µM) or U0126 (10 µM) for 24 h. Values are means ± standard error of three independent experiments performed in triplicate. **(B)** Western blot shows lysates from HKs treated with vorinostat (5 µM) and LY294002 alone (10 µM) or in combination. Ct, control; Vo, vorinostat; LY, LY294002; l+v, LY294002+vorinostat. Figure is representative of two independent experiments with similar results. **(C)** Western blot shows lysates from HKs and SCC25 cells treated with vorinostat (5 µM) for 24 h. Figure is representative of three independent experiments with similar results.

***Supplementary figure 3.*** ***ROS generation correlates with enhanced caspase-dependent cytotoxicity induced by Vorinostat/LY combination in SCC cells****.* **(A, B)** SCC25 cells were treated with vorinostat alone or in combination with LY294002 in the presence or absence of vitamin E (VE) for 24 h, labelled with oxidative-sensitive dye CM-H2DCFDA and analyzed by FACS for increase in FITC fluorescence. Values are means of three independent experiments. **(C)** Viability assay of SCC25 cells subjected to distinct treatments for 24 h with or without vitamin E pre-treatment. Values are means ± standard error of two independent experiments performed in triplicate. **(D)** Viability assay of SCC25 cells subjected to distinct treatments for 24 h in the presence or absence of ZVAD-FMK (100 µM). Values are means ± standard error of three independent experiments performed in triplicate. * indicates *p<*0.05, ** indicates *p*≤0.01 and *** indicates *p*≤0.001 versus respective controls. VE 0.1, vitamin E (0.1 mM); VE 1, vitamin E (1 mM).

***Supplementary figure 4.*** ***Histone hyperacetylation induced by LBH589 and inhibition of AKT phosphorylation induced by BEZ235, BGT226 and BKM120 in SCC25 cells****.* SCC25 cells were treated for 48 hr with distinct concentrations of LBH589, BEZ235, BGT226 or BKM120. Total cellular protein was extracted and samples run on an SDS PAGE gel. S473 AKT phosphorylation (p-AKT), total AKT, (AKT), acetylated histone H3 (ac-H3) and actin were then detected by western blot. Figure is representative of at least 2 independent experiments.

***Supplementary Figure 5. Cytostatic and cytotoxic profile of PI3KI and HDACIs in SCC cell lines.*** SCC cell lines (SCC25 & Cal27) were treated with varying concentrations of LBH589, BEZ235, BGT226 or BKM120 for 48 hr after which proliferation (BrdU) or viability (MTS assay) were measured. Values are means + standard error of two independent experiments performed in triplicate.

***Supplementary Figure 6. Clinically relevant PI3K-AKT-mTOR inhibitors enhance cancer cell specific cytotoxicity induced by LBH589 in Cal 27 cells*.** Cal 27 cells were treated for 48 hs with LBH589 (300 nM), BEZ235 (300 nM), BKM120 (300 nM) and BGT226 (300 nM), alone in combinations. Viability was determined by MTS assay. Values are means ± standard error of three independent experiments performed in triplicate. * indicates *p<*0.05.


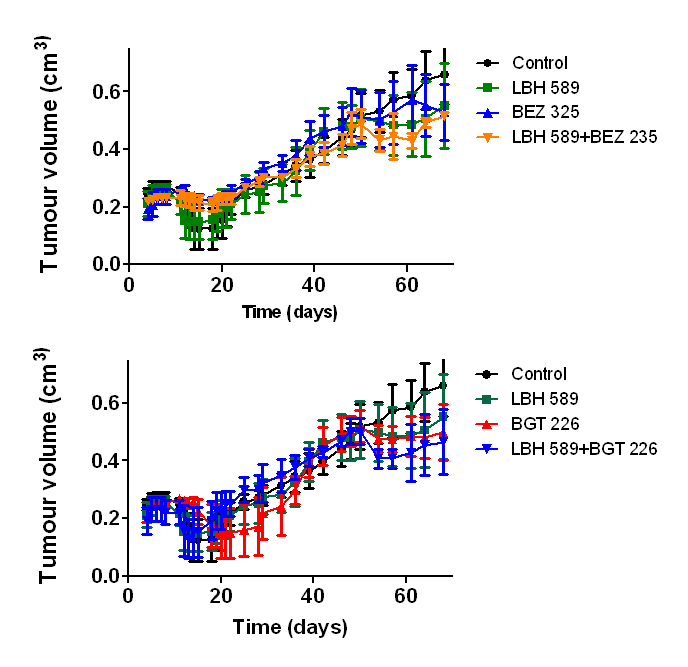


***Supplementary Figure 7. Antitumour properties of LBH589 and PI3K/mTOR/AKT inhibitors in a xenotransplant model of HNSCC***. **(A)** Six week old NOD/SCID mice (groups of 4) were injected with 2.5 x 10^5^ SCC25 cells on day 0. 45 days after injection of cells mice were treated with i) vehicle only, ii) LBH589 (30 mg/kg/day i.p.), iii) BEZ235 (30 mg/kg/day p.o.), iv) BGT226 (10 mg/kg/day p.o.), v) LBH589 (30 mg/kg/day i.p.) + BEZ235 (30 mg/kg/day p.o.), or vii) LBH589 (30 mg/kg/day i.p.) + BGT226 (10 mg/kg/day p.o.). Data presented as mean + sem of individual measurements from 4 mice / group. Dotted line indicates beginning of treatment for distinct groups.
